# Dunglison’s American Medical Library

**Published:** 1841-08

**Authors:** 


### DUNGLISON'S AMERICAN MEDICAL LIBRARY.—NEW SERIES.

We have received the first number of the new series of the Library and Intelligencer. It will be issued, hereafter, once a month, instead of semi-monthly, and at $5 a year, instead of $10. The well earned reputation of the editor, and "the high character it has hitherto sustained will be a guarantee of its future excellence."

